# First-Line Chemo-Immunotherapy for Extensive-Stage Small-Cell Lung Cancer: A United States-Based Cost-Effectiveness Analysis

**DOI:** 10.3389/fonc.2021.699781

**Published:** 2021-06-29

**Authors:** Qiao Liu, Xia Luo, Lidan Yi, Xiaohui Zeng, Chongqing Tan

**Affiliations:** ^1^ Department of Pharmacy, The Second Xiangya Hospital of Central South University, Changsha, China; ^2^ Department of Nuclear Medicine/PET Image Center, The Second Xiangya Hospital of Central South University, Changsha, China

**Keywords:** cost-effectiveness, extensive-stage small-cell lung cancer, atezolizumab, durvalumab, etoposide-platinum

## Abstract

**Objective:**

This study aimed to assess the cost-effectiveness of two recently approved first-line chemo-immunotherapies [atezolizumab combined with etoposide and platinum (AEP) and durvalumab combined with etoposide and platinum (DEP)] for patients with extensive-stage small-cell lung cancer (ES-SCLC) in the United States.

**Material and Methods:**

A Markov model was built to compare the cost and effectiveness of AEP, DEP, and etoposide plus platinum (EP) over a 10-year time horizon. Clinical efficacy and safety data were extracted from the IMpower 133 and CASPIAN trials. Health state utilities were obtained from published literature. Costs were collected from an US payer perspective. Deterministic and probabilistic sensitivity analyses were used to explore the uncertainty bound to model parameters.

**Results:**

For the model cohort of adult patients with treatment-naive ES-SCLC, AEP was associated with marginal improved quality adjusted life years (QALYs) by 0.016 and reduced costs by $5,737 compared with DEP. When comparing the two chemo-immunotherapies with EP chemotherapy, AEP and DEP increased the QALYs by 0.162 QALYs and 0.146, respectively. However, both chemo-immunotherapies were associated with substantially health costs than EP, resulting in ICERs of $382,469 per QALY and $464,593 per QALY, respectively.

**Conclusion:**

In this cost-effectiveness study, first-line AEP represented a dominant treatment strategy compared with DEP. Despite neither first-line AEP nor first-line DEP was cost-effective compared with EP chemotherapy, AEP was able to provide a more efficient balance between incremental cost and QALY than DEP. When new combination therapies with remarkable effect become pivotal in the first-line treatment, the price reduction of these drugs may be essential to achieving cost-effectiveness.

## Introduction

Small cell lung cancer (SCLC) contributes to approximately 14% of all lung malignancies (1, 2), and up to two thirds of patients diagnosed with SCLC are classified as having extensive-stage small-cell lung cancer (ES-SCLC) (3). Over the past few decades, etoposide plus platinum (EP) remained the mainstay of standard-of-care first-line treatment for ES-SCLC, with few alternatives (4–6). Although ES-SCLC is highly sensitive to first-line chemotherapy, almost all cases experience a recurrence within 6 months, resulting in a dismal prognosis with a 5-year survival rate lower than 5% (7, 8). To improve patients’ prognosis and outcomes, immune checkpoint inhibitors (ICIs) in combination with EP chemotherapy has emerged as a new first-line treatment option for ES-SCLC.

Atezolizumab was the first ICI approved by the US Food and Drug Administration (FDA) in March 2019 to combine with EP chemotherapy as a first-line option for treating ES-SCLC (9). The study underpinning this approval was a randomized phase III IMpower 133 trial (ClinicalTrials.gov number:NCT02763579) showing that the combination therapy of atezolizumab and EP chemotherapy significantly improved overall survival (OS) and progression-free survival (PFS) in patients with ES-SCLC compared with the standard-of-care EP chemotherapy (10). Driven by this promising result, there is a growing interest in exploring novel chemo-immunotherapy. At the end of 2019, the randomized phase III trial, CASPIAN (ClinicalTrials.gov number: NCT03043872), demonstrated that adding durvalumab to the first-line EP chemotherapy significantly improve patients’ survival compared with EP chemotherapy (11). Based on these data, durvalumab in combination with EP became the second chemo-immunotherapy approved for the first-line treatment for ES-SCLC (12).

The introduction of chemo-immunotherapy in the first-line setting of ES-SCLC is of great clinical importance and significance, given that a potentially huge population may benefit from the two innovative combination therapies. A total of 235,760 new cases of lung cancers were projected to occur in the United States in 2021 (13), forming a potential beneficiary population of nearly 22,000 ES-SCLC patients. Although the approval of the two chemo-immunotherapies represented a major step forward in providing more successful strategies for the first-line treatment of ES-SCLC, their prohibitive cost cannot be ignored, given the growing demand of providing value-based healthcare in the US (14). Thus, cost-effectiveness studies to assess the clinical benefits and potential financial consequences of an innovative combination therapy are necessary to determine the appropriateness of its widespread use.

Previous US-based studies demonstrated that adding atezolizumab or durvalumab to the first-line EP chemotherapy were associated with higher costs and greater benefits and concluded that the combinations were not a cost-effective choice for ES-SCLC as compared with chemotherapy alone (15, 16). Despite this, these two combination therapies are recommended as the first-line treatment for ES-SCLC over EP chemotherapy alone in the current treatment guidelines (17). However, whether these two approved chemo-immunotherapies are similarly cost-effective, or one is superior to another, remains unclear due to lack of relevant evidence. To answer this question, we conducted this study to compare the cost-effectiveness of atezolizumab combined with etoposide and platinum (AEP) and durvalumab combined with etoposide and platinum (DEP) among ES-SCLC patients from an US payer perspective

## Materials and Methods

This economic evaluation used existing patient data from two published phase III clinical trials (the IMpower 133 trial and the CASPIAN trial) and did not involve human subject research. Therefore, it was deemed exempt from the institutional review board approval. Our study followed the Consolidated Health Economic Evaluation Reporting Standards (CHEERS) reporting guideline.

Using TreeAge Pro 2020 software (TreeAge Software LLC), we constructed a Markov model to compare the long-term health and cost outcomes of patients with ES-SCLC. Three first-line treatment options were evaluated in our model, including two chemo-immunotherapies (AEP and DEP), and the traditional EP chemotherapy. Adding an EP chemotherapy group into the model is because EP chemotherapy is still recommended as a first-line option for ES-SCLC based on the latest national comprehensive cancer network (NCCN) guidelines (17).

### Patients and Treatment

Model patients in the AEP group and the DEP group mirrored the cohorts of participants that were enrolled in the IMpower 133 and CASPIAN trial, respectively (10, 12). We assumed the model patient cohort in the EP group was a combination of two chemotherapy groups in the IMpower 133 and CASPIAN trials. First-line treatment schedule and dosage followed those detailed by the abovementioned two clinical trials. **Supplementary Table 1** in the Supplement provides detail information on each first-line treatment.

After progression, subsequent therapy options for ES-SCLC patients are generally limited, and the current standard-of-care is chemotherapy with topotecan. Considering that other subsequent therapy types are far less used than the topotecan chemotherapy and the specific drugs for subsequent therapies were not detailed in these two clinical trials, we modeled patients as receiving only topotecan as the subsequent therapy. In the IMpower 133 and CASPIAN trials, almost half of patients who exhibited evidence of disease progression were reported to receive a subsequent therapy (51.7% in the AEP group; 42.0% in the DEP group, and a pooled estimated of 51.6% in the EP group) (10, 12). Subsequent topotecan treatment schedule and dosage were given based on the representative clinical trial (18).

### Model Construction

**Figure 1:**
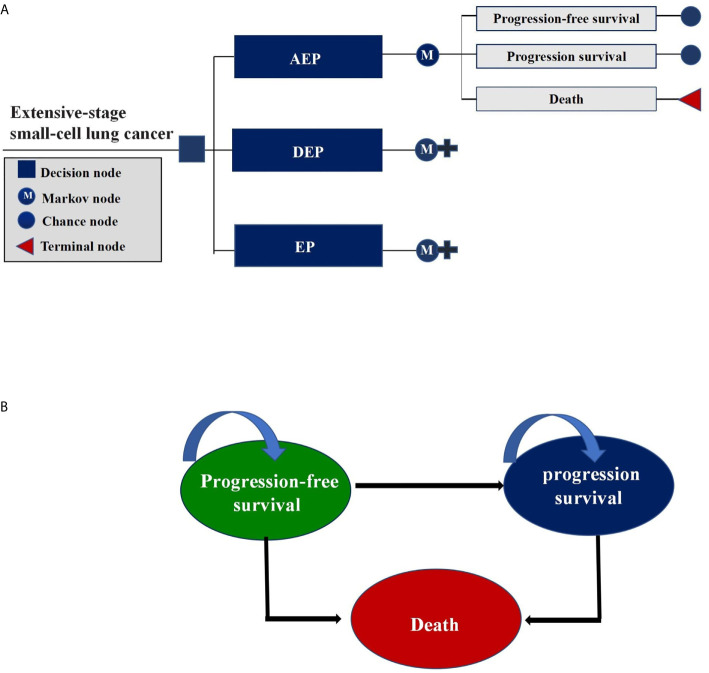
Markov Model. **(A)** Schematics of the decision tree showing 3 treatment strategies compared in our model for patients with extensive-stage small-cell lung cancer; **(B)** Markov state transition model diagram showing 3 health states that represented the process of disease progression. M indicates Markov model.

We constructed a Markov model consisting of three health states in this cost-effectiveness analysis: PFS, progressed survival (PS), and death (**Figure 1**). All ES-SCLC patients entered the model in PFS state and could receive three first-line treatments randomly. In the PS state, patients were considered for topotecan if there was a continued benefit; otherwise, supportive treatment was considered (17). To better accommodate the current clinical practice, patients were assumed to receive palliative care before death.

In view of the clinical treatment plan and the expected overall survival time of ES-SCLC, a 3-week Markov cycle and a 10-year time horizon were chosen for our model to project the cumulative costs and effectiveness in quality adjusted life years (QALYs) for each treatment strategy. Cost-effectiveness was assessed by the incremental cost-effectiveness ratios (ICERs) between treatment strategies under comparison, which reflected the incremental cost for each QALYs gained. In this analysis, ICERs were compared with a willingness-to-pay (WTP) of $100,000 per QALY gained (19), and both costs and effectiveness were discounted at an annual rate of 3%. The Markov model was constructed using TreeAge Pro software (version 2021, https://www.treeage.com/), and parametric survival modeling was performed using R software (version 4.0.4, http://www.r-project.org).

### Transition Probabilities

Transition probabilities were estimated from the IMpower 133 and CASPIAN trials (10, 12). For AEP group, the OS and PFS data over first 2 years were extracted from the Kaplan-Meier (KM) curves using GetData Graph Digitizer software package (version 2.26; http://getdata-graph-digitizer.com/index.php), and best fit with log-logistic survival distribution according to the Akaike’s information criterion (AIC) and Bayesian information criterion (BIC) (**Supplementary Table 2** and **Figure 1**). For DEP group, the log-logistic survival distribution was adjusted using the HRs of OS and PFS for DEP versus AEP generated by network meta-analysis, and the survival rates for DEP were calculated according to the following formula: *S_DEP_* = (*S_AEP_*)*^HR^*. For EP chemotherapy, using the method proposed by Hoyle et al. (20), we recreated two sets of individual patient-level OS and PFS data based on the IMpower 133 trial and the CASPIAN trial, respectively. Then we integrated the two sets of PFS and OS data into the PFS and OS data of EP group in our model. Weibull distribution was used to fit these integrated PFS and OS data (**Supplementary Table 2** and **Figure 1**). The final distribution parameters used to calculate the transition probabilities were outlined in **Table 1**.

**Table 1:** Model Parameters and Assumptions.

Parameters	Baseline value	Ranges	Distribution	Ref
Survival				
Log-logistic survival model for AEP^a^				
OS	θ=0.003072, κ=2.297440	–	–	(10)
PFS	θ=0.008895, κ=2.852489	–	–	(10)
HR for AEP *vs* DEP^b^				
OS	1.04	0.83–1.25	Lognormal	(10, 12)
PFS	1.01	0.81–1.21	Lognormal	(10, 12)
Weibull survival model for EP^c^				
OS	λ=0.016073, γ=1.593409	–	–	(10, 12)
PFS	λ=0.042826, γ=1.712046	–	–	(10, 12)
**Costs**				
Atezolizumab price/mg	7.83	5.87–9.78	Gamma	(21)
Durvalumab price/mg	7.60	5.70–9.50	Gamma	(21)
Etoposide price/mg	1.51	1.13–1.89	Gamma	(21)
Carboplatin price/mg	0.06	0.04–0.07	Gamma	(21)
Topotecan price/mg	12.75	9.56–15.94	Gamma	(21)
Advent event (1st-line atezolizumab plus chemotherapy)	4959.82	3719.87–6199.78	Gamma	(22)
Advent event (1st-line durvalumab plus chemotherapy)	4743.05	3557.29–5928.81	Gamma	(22)
Advent event (1st-line chemotherapy)	6100.94	4508.96–7514.93	Gamma	(22)
Advent event (2nd-line topotecan)	14487.33	10865.50–18109.16	Gamma	(22)
Administration intravenous, first hour	142.55	106.91–178.19	Gamma	(23)
Administration intravenous, additional hour	30.68	23.01–38.35	Gamma	(23)
Monthly physician visit	148.33	111.25–185.41	Gamma	(23)
Three-monthly imaging	122.71	92.03–153.39	Gamma	(23)
Monthly supportive care	637.00	477.75–796.25	Gamma	(24)
Death associated costs	9433.00	7074.75–11791.25	Gamma	(24)
**Utilities**				
PFS	0.673	0.538–0.808	Beta	(15, 16)
PS	0.473	0.378–0.568	Beta	(15, 16)
Disutility for EP	0.112	0.090–0.134	Beta	(25)
Disutility for AEP	0.090	0.072–0.108	Beta	(25)
Disutility for DEP	0.094	0.075–0.113	Beta	(25)
**Others**				
Proportion of subsequent therapy in the atezolizumab plus chemotherapy group	0.517	0.414–0.620	Beta	(10)
Proportion of subsequent therapy in the durvalumab plus chemotherapy group	0.420	0.336–0.504	Beta	(12)
Proportion of subsequent therapy in the chemotherapy group	0.516	0.413–0.619	Beta	(10, 12)
Body surface area (meters^2^)	1.80	1.35–2.25	Gamma	(16)
Creatinine clearance rate(ml/min)	70.00	52.50–87.50	Gamma	(16)

a Theta (θ) and kappa (γ) represented two parameters of log-logistic distribution.

b The OS HR and PFS HR for AEP vs DEP were generated using network meta-analysis.

c Lambda (λ) and gamma (γ) represented two parameters of Weibull distribution.

AEP, atezolizumab combined with etoposide and platinum; DEP, durvalumab combined with etoposide and platinum; EP, etoposide plus platinum; OS, overall survival; PFS, progression-free survival; PS, progressed survival.

PFS projections beyond the 2-year follow-up period were based on the survival distributions selected for the estimated PFS data for first 2 years. OS projections beyond the 2-year follow-up period were based on Surveillance, Epidemiology, and End Results data from 2000 to 2017 for patients with ES-SCLC which allowed the overall survival of ES-SCLC to closely reflect clinical practice (**Supplementary Table 3**) (26).

### Costs and Utilities

Direct medical costs collected from the US payer perspective included drug acquisition and administration costs for the first-line and subsequent therapy, adverse event (AE) management costs, routine follow-up costs, supportive care costs, and death-associated costs.

Drug prices were taken from the October 2020 Centers for Medicare & Medicaid Services (CMS) Average Sales Price Drug Pricing Files (21). For the sake of simplification, the cost of platinum was modeled as the cost of carboplatin in three treatment groups, to take into account the clinical preference for carboplatin over cisplatin. In calculating the drug costs per cycle, the model patient cohort was modeled as a baseline patient with a body surface area of 1.8 m^2^ and a creatinine clearance rate of 70 ml/min (16). Drug administration costs were searched from the CMS Physician Fee Schedule Look-Up Tool (23). For calculating drug administration costs, the durations of EP chemotherapy, and chemo-immunotherapy infusion were modeled as 3 and 4 hour per cycle, respectively. Furthermore, the durations of ICIs were adjusted based on the median treatment cycles to take into account patients’ discontinuations that were not just because of disease progression, but also because of AEs, physician decision, and other reasons (10, 12).

Costs for managing grade III/IV AEs with an incidence of ≥3% were included in the model. The AE management cost for each first-line treatment was estimated by summing the product of the unit cost and the incidence corresponding to each AE. The cost estimation of each AE was sourced from the Healthcare Cost and Utilization Project using diagnosis Code selection for ICD-10 (22). In the model, we assumed that all AEs occur in the first cycle, and the incidence of each AE was quoted from the IMpower 133 and CASPIAN trials (**Supplementary Table 4**). We assumed routine follow-up including a monthly physician visit and a three-monthly imaging examination. Supportive care costs and death-associated costs were derived from published literature (24).

Neither the IMpower 133 trial nor the CASPIAN trial collected information for the quality of life for patients with ES-SCLC. According to the previously published economic evaluation, the PFS and PS health states in our model were assigned the utilities of 0.673 and 0.473, respectively (15, 16). In addition, the utility decrements caused by common grade III/IV AEs associated with treatment were considered in our model (**Supplementary Table 4**) (25).

### Statistical Analysis

To address uncertainty bound to the model parameters, a series of sensitivity analysis were performed. In deterministic sensitivity analyses, model parameters were varied individually to confirm the influence degree of each parameter on the model results. Health state utilities and proportions of subsequent therapy were tested at the upper and lower of their respective 95% CIs. Other parameters were tested within a range of ±25% of baseline values. In probabilistic sensitivity analyses, model parameters were varied simultaneously to verify the robustness of our model. 10,000 Monte Carlo simulations were carried out by randomly sampling model parameters to general 10,000 cost and effectiveness estimates for each treatment strategy. **Table 1** detailed the baseline values, ranges, and distributions of model parameters in the sensitivity analysis.

In addition, we incorporated a scenario analysis in our model, in which the duration of first-line ICIs increased from the median treatment cycles to the treatment cycles of receiving ICIs until disease progression, to explore whether the duration of ICIS had a substantial impact on our results.

## Results

### Base Case Results

The model patient cohort was adult patients with treatment-naive histologically or cytologically documented ES-SCLC. Within a 10-year time horizon, use of AEP was associated with a marginal improvement in QALYs and reduced health care costs of $5,737 compared with use of DEP (**Table 2**). Therefore, AEP was the dominant treatment strategy compared with DEP. The comparisons between the two chemo-immunotherapies and EP chemotherapy demonstrated that adding atezolizumab and durvalumab to the first-line EP chemotherapy gained additional 0.162 and 0.146 QALYs, respectively, which were equivalent to 2 months of perfect health. Due to the improvement in QALYs, AEP, and DEP were associated with substantially greater health care costs than EP chemotherapy, resulting in ICERs of $382,469/QALY and $464,593/QALY, respectively.

**Table 2:** Base case results.

Outcomes	EP	AEP	DEP	Incremental		
				DEP *vs* AEP	AEP *vs* EP	DEP *vs* EP
Cost, $US	24,582	86,655	92,391	5,737	62,073	67,810
QALY	0.578	0.740	0.724	−0.016	0.162	0.146
ICER, $/QALY				Dominated^a^	382,469	464,593

a DEP showed lower effectiveness and higher cost, as compared with the AEP.

EP, etoposide plus platinum; AEP, atezolizumab combined with etoposide and platinum; DEP, durvalumab combined with etoposide and platinum; QALY, quality adjusted life year; ICER, incremental cost-effectiveness ratio.

### Sensitivity Analysis

**Figure 2:**
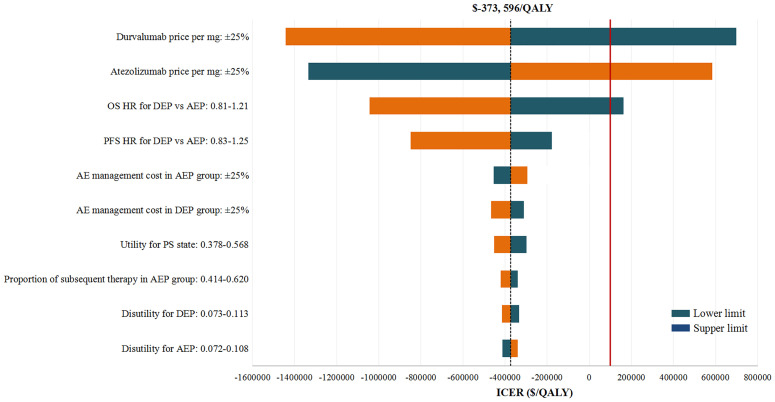
Deterministic Sensitivity Analysis. The red solid line represents the willingness-to-pay threshold of $100,000 used this analysis. The black dotted line represents the incremental cost-effectiveness ratio (ICER) between alternatives under comparison. The top 10 most influential parameters of the ICERs are displayed. AEP indicated atezolizumab combined with etoposide and platinum; DEP, durvalumab combined with etoposide and platinum; QALY, quality adjusted life year; ICER, incremental cost-effectiveness ratio; AE, adverse event; PFS, progression-free survival; PS, progressed survival.

In deterministic sensitivity analyses, when comparing the two chemo-immunotherapies, except the price of durvalumab and atezolizumab, as well as the OS HR of DEP versus AEP, other model parameters failed to change the preferred strategy from AEP to DEP (**Figure 2**). When comparing the two chemo-immunotherapies with EP chemotherapy, first-line AEP and DEP were not cost-effective within the variable range of any tested parameters (**Supplementary Figures 2**, **3**). However, both ICERs were extremely sensitive to the price of the ICIs. A 77% reduction in the price of atezolizumab would allow the ICER for AEP *vs* EP below the WTP threshold of $100,000 per QALY, while a 80% reduction in the price of durvalumab would make the ICER for DEP *vs* EP lower than the WTP threshold of $100,000 per QALY gained.

In probabilistic sensitivity analyses, the comparison of two chemo-immunotherapies suggested that first-line AEP could achieve cost-effectiveness in 100% simulations. When comparing the two chemo-immunotherapies with EP chemotherapy, the probabilities of first-line AEP and DEP being cost-effective were 6.6% and 4.1% at the WTP threshold of $100,000/QALY, respectively (**Supplementary Figure 4**).

The result of our scenario analysis suggested that with the increase of treatment cycles of ICIs, the health costs associated with first-line AEP and DEP increase sharply, but our conclusion had not changed substantially. For example, when we assumed that patients received first-line ICIs until disease progression, the health costs of the fist-line AEP and DEP were $115,595 and $131,987, respectively. However, AEP still dominated DEP, and the ICERs for AEP and DEP were $542,305/QALY and $715,247/QALY, respectively.

## Discussion

Using a Markov model, we estimated the 10-year time horizon costs and effectiveness associated with first-line AEP and DEP by pooling the clinical efficacy and safety data from two large, randomized, phase III clinical trials and collecting costs mainly from the Medicare in 2020. Results of this cost-effectiveness study conducted in United States for patients with ES-SCLC demonstrated that first-line AEP was the dominant treatment strategy compared with DEP, which achieved higher effectiveness at lower health care cost. Furthermore, the current economic evaluation found that the first-line AEP and DEP are not cost-effective compared with EP chemotherapy that was in agreement with previous cost-effectiveness studies (15, 16), but AEP was found to provide a more-efficient balance between the increment cost and QALYs than was DEP.

Sensitivity analyses focusing on uncertainty bound to model parameters confirmed the robustness of our model. The most influential parameters to the model were the price of atezolizumab and durvalumab. We found that the price increase of atezolizumab by more than 10% and the price decrease of durvalumab by more than 9% would allow DEP dominate AEP economically. While the price reduction of atezolizumab by more than 77% and durvalumab by more than 80% would allow the ICERs for AEP *vs* EP and DEP *vs* EP lower than the WTP threshold of $100,000/QALY. After drug prices, the HRs of DEP *vs* AEP had significant effects on the model results, underscoring the necessity of robust head-to-head clinical data. Because changing other parameters had no substantial impact on our results, price decreases for atezolizumab and durvalumab were considered to be the most practical measures for first-line AEP and DEP to achieve cost-effectiveness.

Two previous economic evaluation determined the cost-effectiveness of AEP or DEP versus EP in the first-line setting of ES-SCLC in the United States, and found that the ICER of AEP was $528,810/QALY (0.10 QALY gained at an incremental cost of $52,881) and the ICER of DEP was $355,448 (0.22 QALY gained at an incremental cost of $78,199) over EP, respectively (15, 16). The inconsistency of the ICERs between our study and the previous studies might result from the different long-term survival projections. In the present analysis, the OS data beyond the 2-year follow-up period were derived from Surveillance, Epidemiology, and End Results data from 2000 to 2017, rather than extrapolated directly from the selected survival distribution. In addition, previous studies mainly considered the acquisition and administration costs for first-line drugs, as well as the AE management costs of first-line treatments, while our study also considered the acquisition and administration costs for second-line drug, the AE management costs of second-line treatment, routine follow-up costs, supportive care costs and death-associated costs. Nevertheless, they came to a conclusion similar with our current analysis, that is, neither first-line AEP nor DEP was an optimal strategy from an American perspective.

To our knowledge, this study was the first to compare the cost-effectiveness of two newly approved first-line chemo-immunotherapies for patients with ES-SCLC in the United states. The results of our analysis supported the use of first-line AEP as a cost-effective treatment option for patients with ES-SCLC when compared with DEP. In addition, when compared the current preferred options (AEP and DEP) with the previous preferred option (EP chemotherapy) in the first-line setting for patients with ES-SCLC, the present study pointed out that new combination therapies with remarkable effect allow patients to remain on the costly treatment for relatively long periods, and as a result, their health care costs inevitably soared. Results from the present study had a theoretical value and practical significance for value-based cancer treatments which gives priority to the quality rather than quantity of health care services (27).

This study has several strengths. First, we estimated the 10-year time horizon cost-effectiveness of three first-line treatments for ES-SCLC through economic modeling. In our model, clinical efficacy and safety data were derived from well-conducted phase III clinical trials evidence (the IMpower 133 trial and the CASPIAN trial), and costs were collected from the US payer perspective. As a result, our model can provide a long-term cost and effectiveness projection that can readily translate into clinical practice. Second, the OS data beyond the 2-year follow-up period were derived from Surveillance, Epidemiology, and End Results data from 2000 to 2017 for patients with ES-SCLC (26), which supplement the deficiency that directly extrapolating survival data from the survival distribution used to fit each treatment strategy that may lead to biased long-term OS estimates. Third, to take into account patients’ discontinuation of first-line chemo-immunotherapies that was not solely caused by disease progression (10, 12), but also by other reasons, our model used the median number of cycles that better reflect the time spent on first-line therapy. Furthermore, the result of our scenario analysis suggested that our conclusions had not changed regardless of the increase in treatment cycles. Fourth, our study was comprehensive in that it assessed the only two preferred chemo-immunotherapies recommended by the latest NCCN guidelines for ES-SCLC and the clinical commonly used EP chemotherapy.

This study also has several limitations. First, the comparison between first-line AEP and DEP was indirect because there were no clinical data in one trial to evaluate the two alternatives. There is potential uncertainty here, despite a network meta-analysis was employed in the current study. Second, to simplify the calculation, we assumed that the cost of platinum used across three groups was the cost of carboplatin. On this basis, this analysis likely overestimated the cost of EP chemotherapy because carboplatin is slightly expensive than cisplatin. However, sensitivity analyses showed that varying the cost of carboplatin had almost no influence on the model results. Third, the health state utilities in the model were obtained from the published literature because of the quality-of-life information were not available in both the IMpower 133 trial and CASPIAN trials. Although our findings remained robust over a broad range of health state utilities, the model should be validated against more actual health state utilities.

In conclusion, the economic evaluation between the two first-line chemo-immunotherapies for ES-SCLC suggests that AEP was the dominant treatment strategy compared with AEP. When compared the two first-line chemo-immunotherapies with EP chemotherapy, first-line AEP and DEP are not cost-effective for patients with ES-SCLC, but AEP was able to provide a more-efficient balance between increment cost and QALYs than AEP. When new combination therapies with remarkable effect become pivotal in the first-line treatment, the price reduction of these drugs may be essential for achieving cost-effectiveness.

## Data Availability Statement

The original contributions presented in the study are included in the article/**Supplementary Material**. Further inquiries can be directed to the corresponding authors.

## Author Contributions

XZ and QL had full access to all the data in the study take responsibility for the integrity of the data and the accuracy of the data analysis. Concept and design: QL, XZ, and CT. Acquisition, analysis, or interpretation of data: All authors. Drafting of the manuscript: QL, XZ, and CT. Critical revision of the manuscript for important intellectual content: All authors. Statistical analysis: QL. Obtained funding: QL. Supervision: XZ and CT. All authors contributed to the article and approved the submitted version.

## Funding

This work was supported by the Hunan Provincial Natural Science Foundation [grant 2019JJ50864]; Scientific research project of Hunan Health Commission in 2019 [grant B2019156].

## Conflict of Interest

The authors declare that the research was conducted in the absence of any commercial or financial relationships that could be construed as a potential conflict of interest.

## Supplementary Material

Supplementary Figure 1 Parametric survival distributions fitted for three first-line treatments. AEP indicated atezolizumab combined with etoposide and platinum; DEP, durvalumab combined with etoposide and platinum; EP, etoposide plus platinum.

Supplementary Figure 2 Deterministic Sensitivity Analysis for AEP vs EP. The red solid line represents the willingness-to-pay threshold of $100,000 used this analysis. The black dotted line represents the incremental cost-effectiveness ratio (ICER) between alternatives under comparison. The top 10 most influential parameters of the ICERs are displayed. AEP indicated atezolizumab combined with etoposide and platinum; EP, etoposide plus platinum; QALY, quality adjusted life year; ICER, incremental cost-effectiveness ratio; AE, adverse event; PFS, progression-free survival; PS, progressed survival.

Supplementary Figure 3 Deterministic Sensitivity Analysis for DEP vs EP. The red solid line represents the willingness-to-pay threshold of $100,000 used this analysis. The black dotted line represents the incremental cost-effectiveness ratio (ICER) between alternatives under comparison. The top 10 most influential parameters of the ICERs are displayed. AEP indicated atezolizumab combined with etoposide and platinum; DEP, durvalumab combined with etoposide and platinum; EP, etoposide plus platinum; QALY, quality adjusted life year; ICER, incremental cost-effectiveness ratio; AE, adverse event; OS, overall survival; PFS, progression-free survival; PS, progressed survival.

Supplementary Figure 4 Acceptability curve for the probabilistic sensitivity analysis. AEP indicated atezolizumab combined with etoposide and platinum; DEP, durvalumab combined with etoposide and platinum; EP, etoposide plus platinum; QALY, quality adjusted life year; ICER, incremental cost-effectiveness ratio.
