# Supplementary figures and images for: First-Line Chemo-Immunotherapy for Extensive-Stage Small-Cell Lung Cancer: A United States-Based Cost-Effectiveness Analysis
Source: Front Oncol. 2021 Jun 29;11:699781. doi: 10.3389/fonc.2021.699781 (PMC8276096; doi:10.3389/fonc.2021.699781)

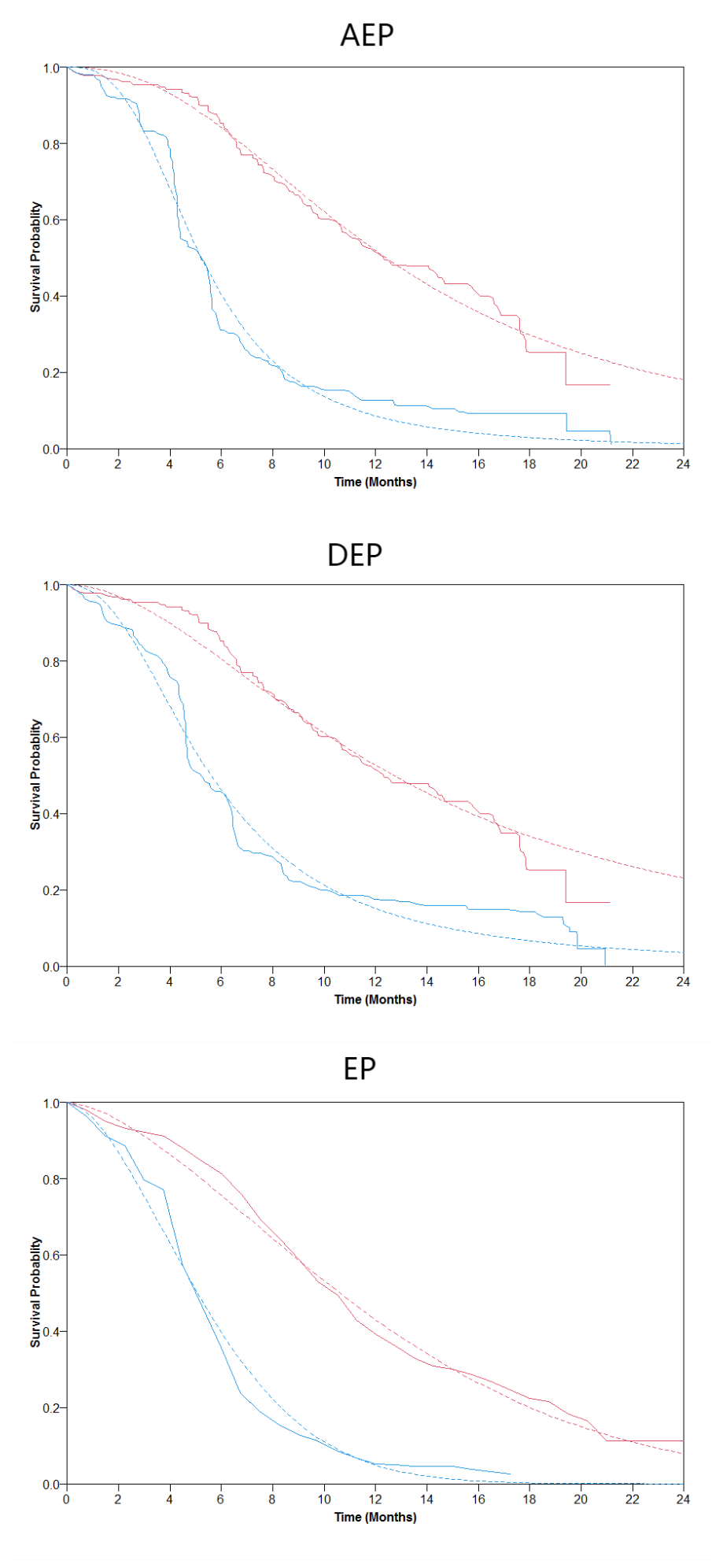

Supplement: Supplementary Figure 1 — Parametric survival distributions fitted for three first-line treatments. AEP indicated atezolizumab combined with etoposide and platinum; DEP, durvalumab combined with etoposide and platinum; EP, etoposide plus platinum. [file Image_1.tif]

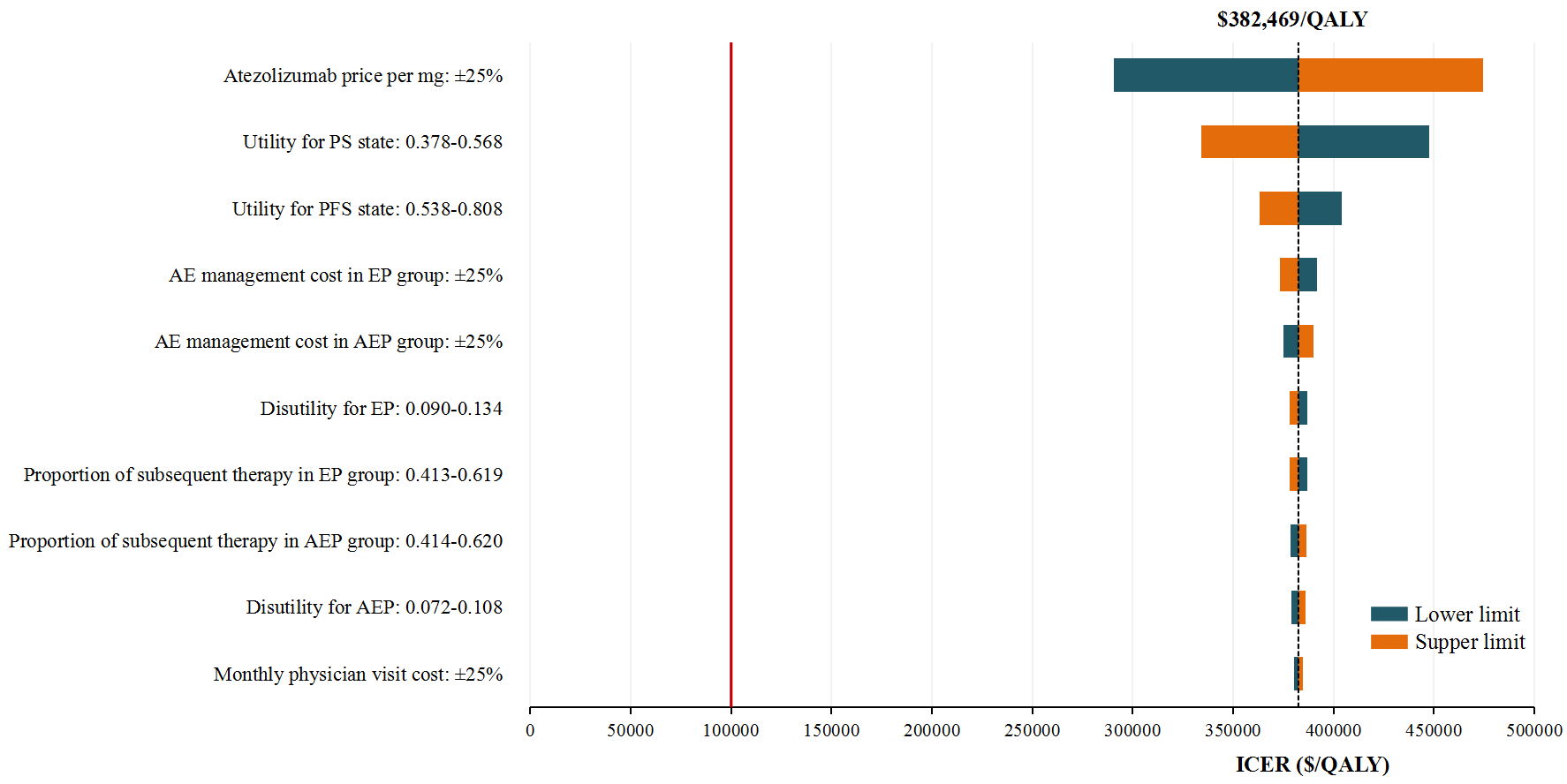

Supplement: Supplementary Figure 2 — Deterministic Sensitivity Analysis for AEP vs EP. The red solid line represents the willingness-to-pay threshold of $100,000 used this analysis. The black dotted line represents the incremental cost-effectiveness ratio (ICER) between alternatives under comparison. The top 10 most influential parameters of the ICERs are displayed. AEP indicated atezolizumab combined with etoposide and platinum; EP, etoposide plus platinum; QALY, quality adjusted life year; ICER, incremental cost-effectiveness ratio; AE, adverse event; PFS, progression-free survival; PS, progressed survival. [file Image_2.tif]

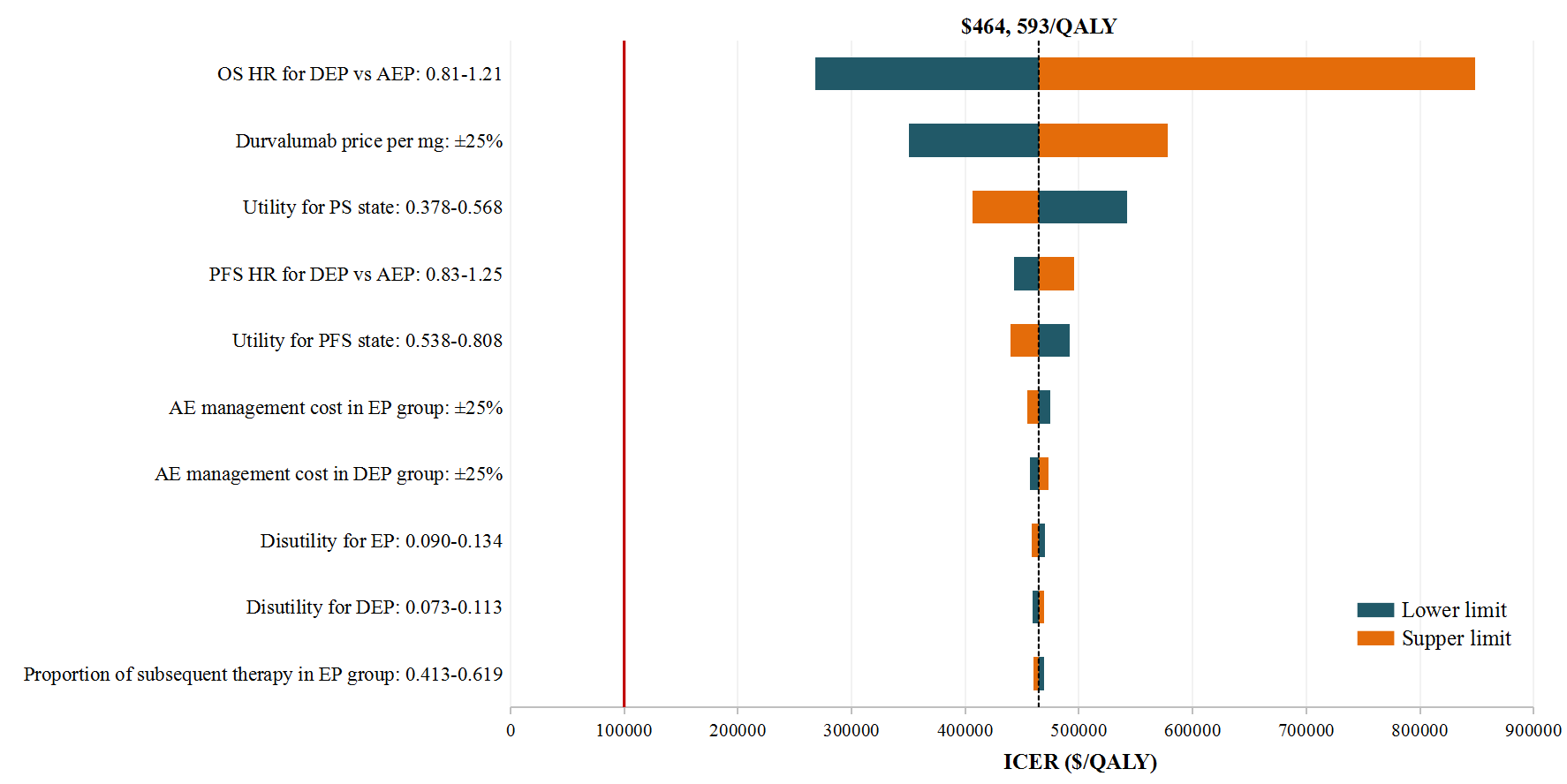

Supplement: Supplementary Figure 3 — Deterministic Sensitivity Analysis for DEP vs EP. The red solid line represents the willingness-to-pay threshold of $100,000 used this analysis. The black dotted line represents the incremental cost-effectiveness ratio (ICER) between alternatives under comparison. The top 10 most influential parameters of the ICERs are displayed. AEP indicated atezolizumab combined with etoposide and platinum; DEP, durvalumab combined with etoposide and platinum; EP, etoposide plus platinum; QALY, quality adjusted life year; ICER, incremental cost-effectiveness ratio; AE, adverse event; OS, overall survival; PFS, progression-free survival; PS, progressed survival. [file Image_3.tif]

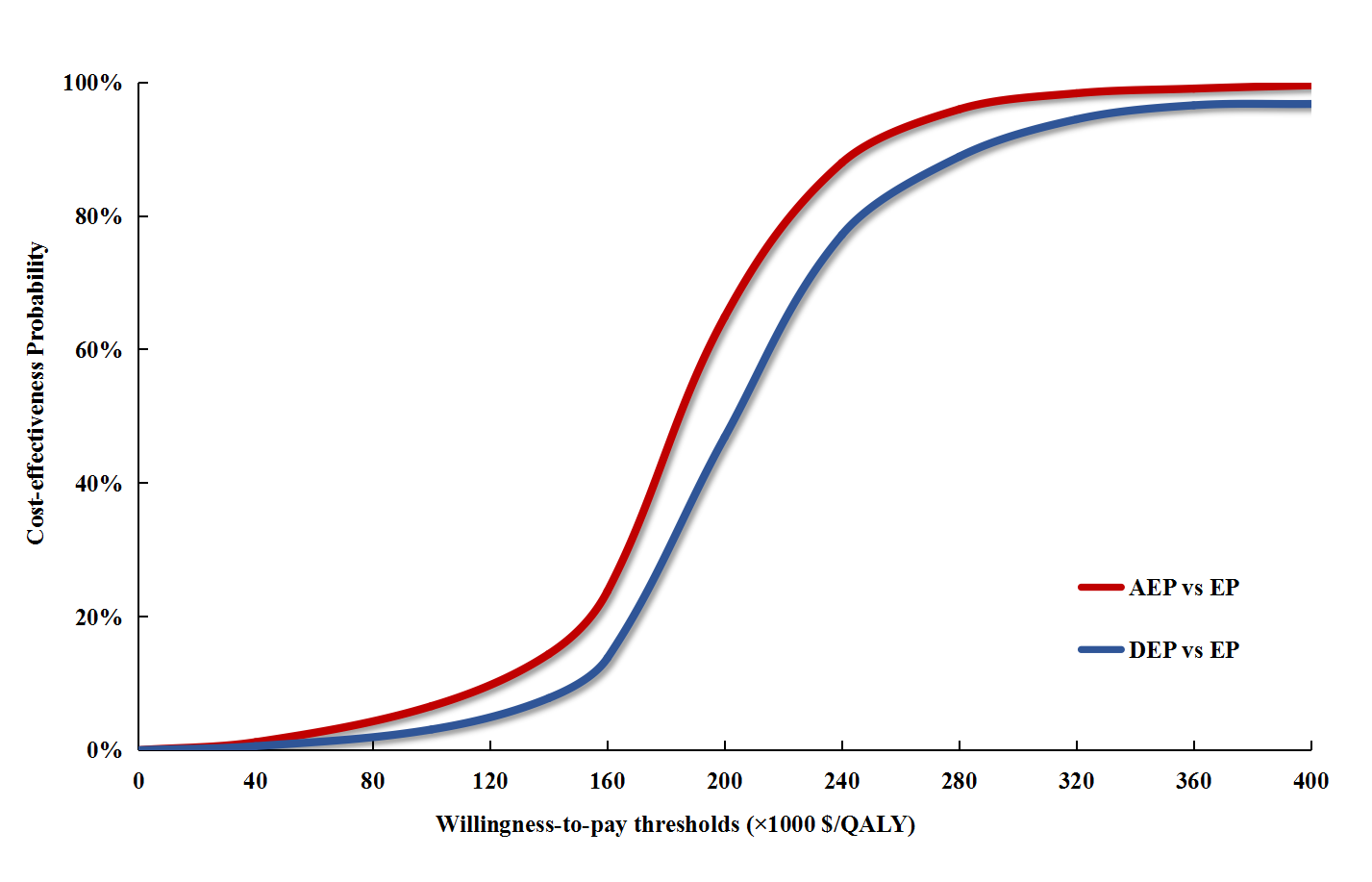

Supplement: Supplementary Figure 4 — Acceptability curve for the probabilistic sensitivity analysis. AEP indicated atezolizumab combined with etoposide and platinum; DEP, durvalumab combined with etoposide and platinum; EP, etoposide plus platinum; QALY, quality adjusted life year; ICER, incremental cost-effectiveness ratio. [file Image_4.tif]
