# Supplementary material for: First-Line Chemo-Immunotherapy for Extensive-Stage Small-Cell Lung Cancer: A United States-Based Cost-Effectiveness Analysis
Source: Front Oncol. 2021 Jun 29;11:699781. doi: 10.3389/fonc.2021.699781 (PMC8276096; doi:10.3389/fonc.2021.699781)
Supplement: Supplementary file 5 [file Table_1.docx]

Table 1. First-line treatment regimens.

| **Regimens** | **Dose Sizes** | **Infusion Timing** | **Median doses** |
| --- | --- | --- | --- |
| first-line AEP | atezolizumab,1200mg; | four 3-week cycles; followed by maintenance atezolizumab every 3 weeks | atezolizumab,7; |
|  | etoposide, 100mg/m^2^_;_ |  | etoposide, 12_;_ |
|  | carboplatin, AUC 5.0mg/ml/min |  | carboplatin, 4 |
| first-line DEP | durvalumab,1500mg; | four 3-week cycles; followed by maintenance durvalumab every 4 weeks | durvalumab,7; |
|  | etoposide, 100mg/m^2^_;_ |  | etoposide, 12_;_ |
|  | carboplatin, AUC 5.0mg/ml/min |  | carboplatin, 4 |
| first-line EP | etoposide, 100mg/m^2^; | Four to six 3-week cycles | etoposide, 12_;_ |
|  | carboplatin, AUC 5.0mg/ml/min |  | carboplatin, 4 |

*AEP, atezolizumab combined with etoposide and platinum; DEP, durvalumab combined with etoposide and platinum; EP, etoposide plus platinum.*
