# Supplementary material for: First-Line Chemo-Immunotherapy for Extensive-Stage Small-Cell Lung Cancer: A United States-Based Cost-Effectiveness Analysis
Source: Front Oncol. 2021 Jun 29;11:699781. doi: 10.3389/fonc.2021.699781 (PMC8276096; doi:10.3389/fonc.2021.699781)
Supplement: Supplementary file 6 [file Table_2.docx]

Table 2. Parametric survival distributions fitted for three first-line treatments.

| **Distribution** | **AEP** | | | | **EP** | | | |
| --- | --- | --- | --- | --- | --- | --- | --- | --- |
|  | OS | | PFS | | OS | | PFS | |
|  | AIC | BIC | AIC | BIC | AIC | BIC | AIC | BIC |
| Exponential | -359 | -353 | -245 | -239 | -66 | -63 | -38 | -35 |
| Weibull | -735 | -725 | -385 | -376 | -159 | -155 | -103 | -99 |
| Log-normal | -126 | -121 | -460 | -451 | -126 | -122 | -94 | -91 |
| Log-logistic | -741 | -731 | -493 | -484 | -141 | -137 | -88 | -84 |

*AEP, atezolizumab combined with etoposide and platinum; EP, etoposide plus platinum; OS, overall survival; PFS, progression-free survival.*
