# Supplementary material for: First-Line Chemo-Immunotherapy for Extensive-Stage Small-Cell Lung Cancer: A United States-Based Cost-Effectiveness Analysis
Source: Front Oncol. 2021 Jun 29;11:699781. doi: 10.3389/fonc.2021.699781 (PMC8276096; doi:10.3389/fonc.2021.699781)
Supplement: Supplementary file 7 [file Table_3.docx]

Table 3. SEER survival statistic: ES-SCLC (2000-2017).

| **Months** | **survival estimates** | **Months** | **survival estimates** | **Months** | **survival estimates** | **Months** | **survival estimates** |
| --- | --- | --- | --- | --- | --- | --- | --- |
| 25 | 6.56% | 49 | 2.93% | 73 | 2.15% | 97 | 1.67% |
| 26 | 6.18% | 50 | 2.88% | 74 | 2.12% | 98 | 1.66% |
| 27 | 5.87% | 51 | 2.84% | 75 | 2.11% | 99 | 1.66% |
| 28 | 5.54% | 52 | 2.80% | 76 | 2.08% | 100 | 1.66% |
| 29 | 5.20% | 53 | 2.77% | 77 | 2.07% | 101 | 1.66% |
| 30 | 4.92% | 54 | 2.74% | 78 | 2.05% | 102 | 1.65% |
| 31 | 4.74% | 55 | 2.69% | 79 | 2.04% | 103 | 1.60% |
| 32 | 4.56% | 56 | 2.66% | 80 | 2.02% | 104 | 1.57% |
| 33 | 4.41% | 57 | 2.60% | 81 | 1.99% | 105 | 1.57% |
| 34 | 4.25% | 58 | 2.60% | 82 | 1.99% | 106 | 1.55% |
| 35 | 4.10% | 59 | 2.58% | 83 | 1.97% | 107 | 1.55% |
| 36 | 4.02% | 60 | 2.55% | 84 | 1.95% | 108 | 1.53% |
| 37 | 3.90% | 61 | 2.53% | 85 | 1.91% | 109 | 1.49% |
| 38 | 3.73% | 62 | 2.46% | 86 | 1.90% | 110 | 1.46% |
| 39 | 3.63% | 63 | 2.45% | 87 | 1.88% | 111 | 1.45% |
| 40 | 3.52% | 64 | 2.40% | 88 | 1.87% | 112 | 1.45% |
| 41 | 3.49% | 65 | 2.38% | 89 | 1.84% | 113 | 1.39% |
| 42 | 3.42% | 66 | 2.35% | 90 | 1.81% | 114 | 1.36% |
| 43 | 3.37% | 67 | 2.33% | 91 | 1.81% | 115 | 1.33% |
| 44 | 3.32% | 68 | 2.32% | 92 | 1.78% | 116 | 1.33% |
| 45 | 3.25% | 69 | 2.26% | 93 | 1.76% | 117 | 1.32% |
| 46 | 3.12% | 70 | 2.21% | 94 | 1.74% | 118 | 1.27% |
| 47 | 3.07% | 71 | 2.19% | 95 | 1.71% | 119 | 1.25% |
| 48 | 3.02% | 72 | 2.16% | 96 | 1.67% | 120 | 1.24% |

*SEER, Surveillance, Epidemiology, and End Results;ES-SCLC, extensive-stage small-cell lung cancer.*
