# Supplementary material for: First-Line Chemo-Immunotherapy for Extensive-Stage Small-Cell Lung Cancer: A United States-Based Cost-Effectiveness Analysis
Source: Front Oncol. 2021 Jun 29;11:699781. doi: 10.3389/fonc.2021.699781 (PMC8276096; doi:10.3389/fonc.2021.699781)
Supplement: Supplementary file 8 [file Table_4.docx]

Table 4. Proportion, costs and disutility of common grade III/IV AEs associated with treatment.

| AEs | Proportion (%) | | | Cost per event ($) | Disutility |
| --- | --- | --- | --- | --- | --- |
|  | EP | AEP | DEP |  |  |
| Neutropenia | 29.4% | 22.7% | 24.2% | 12365 | 0.35 |
| Anaemia | 15.6% | 14.1% | 9.1% | 7248 | /^b^ |
| Thrombocytopenia | 8.6% | 10.1% | 5.7% | 11198 | /^b^ |
| Hyponatraemia | 1.5% | 0.0% | 3.8% | 7389 | /^b^ |
| Pneumonia | 1.9% | 0.0% | 1.9% | 9057 | /^b^ |
| Fatigue | 0.8% | 1.5% | 2.0% | Not considered in the model^a^ | 0.29 |
| Diarrhea | 0.8% | 2.0% | 1.1% | Not considered in the model^a^ | 0.22 |
| Vomiting | 2.3% | 1.0% | 0.0% | Not considered in the model^a^ | 0.20 |
| Hypertension | 0.2% | 0.0% | 3.0% | Not considered in the model^a^ | 0.03 |
| Estimated AEs Costs and disutility | | | |  |  |
| AEs cost for EP, $ | | | | 6011.94 |  |
| AEs cost for AEP, $ | | | | 4959.82 |  |
| AEs cost for DEP, $ | | | | 4743.05 |  |
| AEs disutility for EP | | | |  | 0.112 |
| AEs disutility for AEP | | | |  | 0.090 |
| AEs disutility for DEP | | | |  | 0.094 |

*AEs, advance events; EP, etoposide plus platinum; AEP, atezolizumab combined with etoposide and platinum; DEP, durvalumab combined with etoposide and platinum.*

*^a^Our model considered the management costs of grade III/IV AEs with an incidence of ≥3% were included in the model, therefore, the costs for fatigue, diarrhea, vomiting, hypertension were excluded.*

*^b^The disutilities with regard to anaemia,thrombocytopenia, hyponatraemia, pneumonia were not reported.*
